# The equivalent internal orientation and position noise for contour integration

**DOI:** 10.1038/s41598-017-13244-z

**Published:** 2017-10-12

**Authors:** Alex S. Baldwin, Minnie Fu, Reza Farivar, Robert F. Hess

**Affiliations:** McGill Vision Research, Department of Ophthalmology, McGill University, Montreal, Quebec Canada

## Abstract

Contour integration is the joining-up of local responses to parts of a contour into a continuous percept. In typical studies observers detect contours formed of discrete wavelets, presented against a background of random wavelets. This measures performance for detecting contours in the limiting external noise that background provides. Our novel task measures contour integration without requiring any background noise. This allowed us to perform noise-masking experiments using orientation and position noise. From these we measure the equivalent internal noise for contour integration. We found an orientation noise of 6° and position noise of 3 arcmin. Orientation noise was 2.6x higher in contour integration compared to an orientation discrimination control task. Comparing against a position discrimination task found position noise in contours to be 2.4x lower. This suggests contour integration involves intermediate processing that enhances the quality of element position representation at the expense of element orientation. Efficiency relative to the ideal observer was lower for the contour tasks (36% in orientation noise, 21% in position noise) compared to the controls (54% and 57%).

## Introduction

The perception of lines and edges in the outside world requires the visual system to “join-up” local responses to points along those lines and edges into contours. The contour integration task introduced by Field *et al*.^1^ has the observer detect a contour composed of wavelets in a background of randomly-scattered wavelets. They explained their results with an “association field” model. In that model responses to individual wavelets are linked to form a representation of the contour. The linking is governed by rules concerning the position and orientation of each wavelet with respect to its neighbours. These rules are consistent with what would be expected based on statistical properties of edges found in natural images^2^. This contours-in-noise paradigm has been adapted for use in many subsequent studies^3–7^. For a recent review, see Hess *et al*.^8^.

Here we perform a noise-masking study for contour integration. This involves measuring the effect of different levels of external noise (added to the stimulus on the screen) on performance. From this one can obtain the equivalent internal noise (representing the quality of the input to the process that solves the task) and the calculation efficiency (better processing strategies applied to the input give higher efficiencies, approaching the ideal observer which uses the best possible strategy). One important aspect of the standard contour paradigm is that performance is largely determined by the background noise field^2,9^. Without this background, it would be trivial to detect the presence of the contour. For this reason, it cannot be adapted for use in a noise masking experiment to measure equivalent *internal* noise. Although one could obtain a noise-masking function by adding noise to the contours in this task, fitting an equivalent internal noise parameter would simply quantify the amount of external noise introduced by the background noise field. It would not be possible to break through that other external noise source to measure the internal noise of the mechanism in the brain that performs contour integration. One can consider this in the context of the ideal observer (which achieves optimal performance on the task). A good task to use for an equivalent noise study is one in which the ideal observer is capable of perfect performance when the external noise is zero.

**Figure 1:**
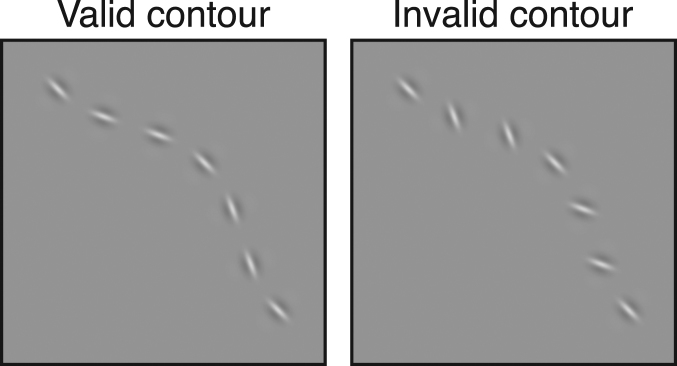
Examples of the valid and invalid contour stimuli used in our experiments. The modulation amplitude (*A*) determining the curvature of the contour is set to 0.25.

To enable the measurement of equivalent noise, we developed a novel task. It is based on the idea that a contour is defined by the conjoined positions and orientations of the parts that compose it. We propose that the visual system integrates contours by finding an appropriate conjunction of position and orientation information^10^. Therefore, we devised a task where the observer must discriminate between two types of stimuli. The stimuli feature sets of wavelets whose position and orientation information form either valid or invalid contours (Fig. 1). For the valid stimulus, we give each wavelet a position and orientation that describes a smooth contour. In the “invalid” stimulus the orientations of the wavelets are flipped so that they would be appropriate for a contour curving in the opposite direction. This gives our stimuli two crucial properties. Firstly, it is not possible to use the position or orientation information alone from the contour to decide if it is valid. The observer is required to combine position and orientation information to solve the task. On this basis we argue that our task tests contour integration. Secondly, our stimuli are deterministic. Our basic task does not rely on the addition of any random noise to make it difficult. The ideal observer could achieve perfect performance, and the point at which the noise has an effect on threshold for our human observers reflects the internal noise of their contour integration process (see below).

The discrimination shown in Fig. 1 is easy. To make the task difficult one can reduce the curvature of the contour by using a smaller amplitude (*A*) in the cosine function that determines its shape. In the previous contours-in-noise task curved contours were more difficult to locate in the noise background. In our task, it is *straighter* contours that make the discrimination of good continuation more difficult. We used a four-alternative forced-choice task where single contours were briefly presented at 2.8 degrees of visual angle from fixation in four quadrants. Observers had to respond with which of the four was the valid contour. Control experiments allow us to compare performance in this task against a non-contour task based on discriminating orientation or position independently. Further details are provided in our methods section. Subjectively, the contour task itself is simple to perform. When the valid contour is detectable it usually “pops out” in an obvious way (giving a smooth continuous impression). This helps observers quickly learn the task.

**Figure 2:**
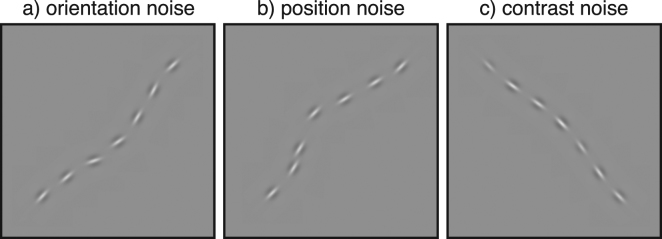
Examples of the different types of noise stimuli used in our experiments. The amplitude of the curvature of the contour for each case is approximately twice that required for threshold performance: (**a**) 0.25, (**b**) 0.25, (**c**) 0.18.

Having established our basic task, we now extend it to measure noise-masking functions. We added three types of noise to our contours: (a) orientation noise that added a random rotation to each of the wavelets in both the valid and invalid contours, (b) position noise implemented by adding a random positional offset to each wavelet, and (c) contrast noise implemented by adding a random contrast jitter to each wavelet. These are shown in Fig. 2, with the contour curvatures at twice that required for threshold performance. Measuring thresholds in different levels of this external (stimulus) noise gives a noise-masking function. From this one can determine the equivalent internal noise in each domain. This is found by seeing how much external noise must be added to the stimulus before performance changes.

Noise masking functions can be interpreted using the Linear Amplifier Model^11^ (LAM). The signal to noise ratio (*d*′) is

1 $$d^{\prime} =\frac{\beta A}{\sqrt{{\sigma }_{{\rm{ext}}}^{2}+{\sigma }_{{\rm{int}}}^{2}}},$$

where *σ*_ext_ is the standard deviation of the external noise added to the stimulus, *σ*_int_ is the standard deviation of the *equivalent* internal noise in the visual system, and *β* represents the efficiency of the processing performed on the input. The amplitude at threshold (*A*_threshold_) can be found by solving for *A* when *d*′ = 1

2 $${A}_{{\rm{threshold}}}=\frac{\sqrt{{\sigma }_{{\rm{ext}}}^{2}+{\sigma }_{{\rm{int}}}^{2}}}{\beta }\mathrm{.}$$

From Eq. (2) one can see that when ${\sigma }_{{\rm{i}}{\rm{n}}{\rm{t}}}\gg {\sigma }_{{\rm{e}}{\rm{x}}{\rm{t}}}$ behaviour will be determined by the internal noise in the system (and the efficiency), and so thresholds will not be affected by the external noise. Once that noise level increases and ${\sigma }_{{\rm{int}}}\ll {\sigma }_{{\rm{ext}}}$ however, the behaviour will instead be driven by that external noise level (and efficiency). This results in a roughly linear increase in *A*_threshold_ as ${\sigma }_{{\rm{ext}}}$ increases. This model was first derived to explain results from contrast detection studies, originally in white Gaussian noise^11–13^, (although later studies have suggested that other types of contrast noise may be more useful, ^14,15^). Since then however, the method and the model have also been applied to texture^16^, motion^17^, and stereoacuity^18^. When applied in this broader sense, the equivalent internal noise ${\sigma }_{{\rm{int}}}$ is a measure of the quality of the input used to perform the task. Its value is affected by intrinsic noise in the visual system, and by input gain or nonlinearities. Its units match those of the external noise, allowing comparisons to be made between tasks.

The efficiency parameter *β* indicates how well the visual system makes use of the noisy input information. For example, in our task if the observers ignored all but a pair of wavelets from each contour when determining which was the valid one (discarding the information available from those other wavelets) this would be inefficient compared to using all the wavelets. In tasks where ideal observer performance has been established efficiency can be measured on an absolute scale relative to that ideal observer. Otherwise, relative efficiency can be compared between observers or conditions that use the same task. In a previous study, Bex *et al*.^19^ have made such comparisons using a modified version of the standard contour task. The standard deviation of the orientation noise added to the contour in the background noise field was varied to measure a psychometric function (perpendicular to the measurements made in this study). This gave a measure of relative efficiency for contour integration.

It is worth noting that previous studies^11^ have presented *calculation efficiency* as $k={(d^{\prime} /\beta )}^{2}$. For any *d*′ this *k* has an inverse square relationship with *β*, the parameter we use to represent efficiency in our fitting. The squaring in the calculation of *k* (or in *η* when it is being calculated as efficiency relative to the ideal observer, ^20,21^) usually has the role of defining efficiency in terms of contrast energy. For the modulation amplitude of our contours this would not have a clear meaning, so by working with *β* as our efficiency parameter we avoid this confusion. Using *β* gives us the vertical offset between noise masking functions, with which we show the ratio between human and ideal performance. If desired, the log_2_ *β* we present in this study can be converted to log_2_ *k* by multiplying them by −2 (and relative log efficiencies should simply be doubled).

For our task, sufficient orientation or position noise should impair performance. This is because the task requires the observer to make use of both of these features. At the end of this paper we develop an ideal observer model to demonstrate how each of these types of noise should make the contour task more difficult. With our contrast noise, we explored the possibility that the contour “code” is multiplexed with the contrast signal. Previous studies have found that collinear arrangements of wavelets reduce their contrast threshold^22,23^. Although some of this effect can be attributed to uncertainty-reduction^24^, there appears to be a small collinear facilitation effect beyond this^25,26^. There is further evidence from neurophysiology that firing rates in V1 are modulated by context of this type^27^. In this case one might predict that adding noise to this code (by randomising the wavelet contrasts) could impair contour integration performance. This question has been investigated previously^28^, however that study used the contours-in-noise approach. The external noise introduced by the random background may have overwhelmed the impairment from the contrast randomisation. We use our new task to take another look at this question, alongside our investigation of the equivalent orientation and position noise.

## Results

**Figure 3:**
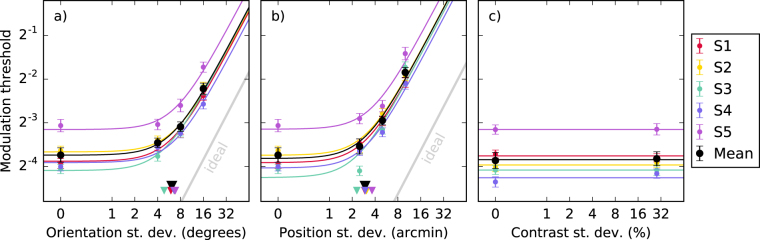
Thresholds (*d*′ = 1) for detecting contours perturbed by the three types of noise shown in Fig. 2. For individual observers the standard errors are calculated over the bootstrap samples. For the mean, standard error is calculated over the observers. Parameters of the LAM fits shown in panels (a,b) are provided in Tables 1 and 2. The triangles on the *x*-axis indicate the equivalent internal noise ${\sigma }_{{\rm{int}}}$. The grey line indicates the efficiency of the ideal observer. In panel (c) a horizontal line is fit to the two data points (0% and 28% contrast noise) to demonstrate that there is no difference between the thresholds.

Noise masking functions obtained from our five observers are shown by the coloured points in Fig. 3. For four out of our five observers, performance was similar between them. The remaining observer (S5) exhibited higher thresholds in all conditions. The mean across the five observers is shown in black. For the orientation and position conditions (Fig. 3a,b) we find that the noise masking functions follow the standard shape. They are initially flat until a critical external noise level is reached, at which point the thresholds increase in proportion to the standard deviation of the masking noise. For the contrast condition (Fig. 3c) the masking noise does not result in any threshold elevation. This shows that randomising the contrast of the wavelets forming the contours had no effect on performance.

**Table 1:** Parameters of the LAM fits to the orientation noise data shown in Fig. 3a.

Observer	log_2_ *β* − log_2_ *β*_ideal_	log_2_ *σ*_int_ (degrees)	RMSe
S1	−1.38 ± 0.11	2.6 ± 0.2	0.05
S2	−1.43 ± 0.10	2.7 ± 0.1	0.12
S3	−1.46 ± 0.09	2.3 ± 0.1	0.05
S4	−1.20 ± 0.12	2.7 ± 0.2	0.08
S5	−1.91 ± 0.12	2.8 ± 0.2	0.09
Mean	−1.48 ± 0.12	2.6 ± 0.1	0.04

For individual observers, the standard error of the bootstrapped parameters is shown. For the mean the standard error is calculated over the observers. In linear units, the mean equivalent internal noise is 6.1°. The efficiency is 36% of the ideal observer.

**Table 2:** Parameters of the LAM fits to the position noise data shown in Fig. 3b. For further details see Table 1. In linear units, the mean equivalent internal noise is 3.0 arcmin. The efficiency is 21% of the ideal observer.

Observer	log_2_ *β* − log_2_ *β*_ideal_	log_2_ *σ*_int_ (arcmin)	RMSe
S1	−2.16 ± 0.09	1.6 ± 0.2	0.03
S2	−2.26 ± 0.09	1.6 ± 0.1	0.12
S3	−2.17 ± 0.08	1.2 ± 0.1	0.30
S4	−2.02 ± 0.10	1.6 ± 0.2	0.10
S5	−2.61 ± 0.12	1.9 ± 0.2	0.16
Mean	−2.25 ± 0.10	1.6 ± 0.1	0.11

We compared thresholds for detecting inwardly and outwardly inflected contours by splitting the data into those two sets. We fitted new psychometric functions and performed a two-way within-subjects ANOVA (factors of inflection direction and noise condition) in *R* ^29^. We found that on average thresholds were 36% higher for detecting outwardly inflected contours. This difference was significant (${F}_{\mathrm{1,4}}=46.06$, $p < 0.01$) but did not interact with noise condition (${F}_{\mathrm{7,28}}=0.68$, $p=0.69$). We performed a similar analysis to investigate whether there was a variation in sensitivity between different target locations. We split the data into quadrants, fitted psychometric functions, and performed a two-way within-subjects ANOVA (factors of quadrant and noise condition). We found no significant effect of quadrant (${F}_{\mathrm{3,12}}=0.36$, $p=0.79$) and no significant interaction between noise condition and quadrant (${F}_{\mathrm{21,84}}=1.10$, $p=0.36$).

The solid lines in Fig. 3a,b show fits of the LAM (Eq. (2)) to the data. Fitting was performed in Python using the fmin function from the SciPy library^30^. This minimised the root-mean-square error (RMSe) between the data and the model prediction (both log-transformed). The details of these fits are shown in Tables 1 and 2. The values of the equivalent internal noise parameters are shown by the triangles in Fig. 3a,b. The five observers were quite consistent with each other. They had fitted equivalent internal orientation noise values of between 5° and 7°, and position noise of between 2 and 4 arcmin. Efficiencies are calculated relative to the ideal observer. Within each condition the efficiency was similar for all observers except for S5, who had lower efficiency in both conditions. The efficiency for the orientation condition was higher than that for the position condition (36% vs. 21%). This indicates that our observers are able to make better use of the information extracted from the orientation noise stimuli than the position noise stimuli.

To control for the sensitivity for discriminating fine position and orientation information at our target locations, we tested five observers on additional non-contour tasks. In the orientation control, observers had to indicate which quadrant contained a wavelet that was rotated. This was done in different levels of orientation noise. In the position control, the task was the same but with a position shift and positional noise. These tasks were chosen with the aim of measuring the position and orientation noise at the level at which the features of individual wavelets are detected. They were designed in such a way that processing strategies involving collinearity cannot solve the task. Our ideal observer models allow us to make direct comparisons between the efficiencies and equivalent internal noises we measure for our contour and control tasks.

**Figure 4:**
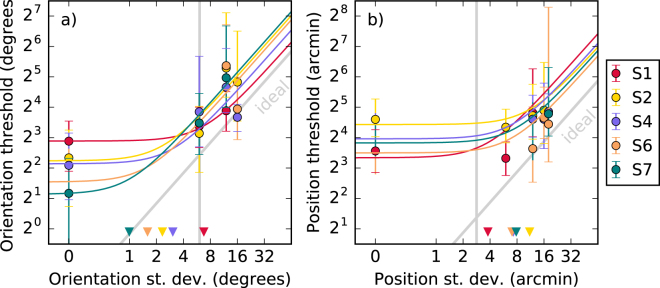
Thresholds (*d*′ = 1) from control tasks, detecting orientation and position shifts in single wavelets. Error bars show standard error calculated over bootstrap samples. LAM fit parameters are shown in Tables 3 and 4. Vertical grey lines show average noise from the contour task, which can be compared against triangles showing the equivalent noise from this task. Diagonal grey lines indicate the efficiency of the ideal observer.

Subjectively, observers found the control tasks more difficult than the contour task. Results are shown in Fig. 4, where one can see that performance was also far more variable between observers. From the triangles one can see that the range of equivalent noise values found is much wider than that seen in the contour task. Tables 3 and 4 show the fitted parameter values with bootstrapped standard errors. These equivalent noise measurements can be compared directly against those from the contour task. We find that the equivalent internal orientation noise in our contour task is 2.6× higher than that for making an orientation judgement on a single wavelet. On the other hand, the equivalent internal noise for the position task is 2.4× *lower*. Efficiency relative to the ideal observer was higher than for the contour tasks. For the orientation control the efficiency was 54%, for the position it was 46%.

**Table 3:** Parameters obtained by performing LAM fits to the data collected in the orientation control experiment (Fig. 4a).

Observer	log_2_ *β* − log_2_ *β*_ideal_	log_2_ *σ*_int_ (degrees)	RMSe
S1	−0.29 ± 0.53	2.8 ± 0.8	0.04
S2	−1.17 ± 0.41	1.2 ± 0.7	0.45
S4	−0.69 ± 0.44	1.6 ± 0.9	0.58
S6	−1.03 ± 0.38	0.7 ± 0.9	0.75
S7	−1.30 ± 0.44	0.0 ± 1.1	0.21
Mean	−0.90 ± 0.18	1.2 ± 0.5	

In linear units, the mean equivalent internal noise is 2.4°. The efficiency is 54% of the ideal observer.

**Table 4:** Parameters obtained by performing LAM fits to the data collected in the position control experiment (Fig. 4b).

Observer	log_2_ *β*	log_2_ *σ*_int_ (arcmin)	RMSe
S1	−1.54 ± 0.51	1.9 ± 0.6	0.37
S2	−1.09 ± 0.86	3.5 ± 0.9	0.12
S4	−1.22 ± 0.54	2.9 ± 0.8	0.09
S6	−0.81 ± 0.77	2.8 ± 1.0	0.28
S7	−0.98 ± 0.75	3.0 ± 0.8	0.18
Mean	−1.13 ± 0.12	2.8 ± 0.3	

In linear units, the mean equivalent internal noise is 7.0 arcmin and the efficiency is 46%.

## Discussion

This novel paradigm provides a new approach, allowing investigation of contour integration at threshold. Applying external noise allows us to measure the equivalent noise in the mechanism responsible for contour integration. Previous studies using similar stimuli to measure contrast detection thresholds have found an interaction between collinearity and contrast processing^22–26^. In line with previous studies that used a contour task however^7,28^, we find that contrast noise does not interfere with contour integration. For orientation and position we are able to measure an equivalent internal noise. These values reflect the quality of the information at the processing level at which contour integration is performed. We were also able to measure efficiency relative to the ideal observer, indicating how effectively the observers made use of that noisy information.

The equivalent internal noise values we measure in our contour task are compared against those from two control tasks (one for orientation, and one for position). Although these tasks feature a different number of wavelets compared to our contour task, this should not affect the equivalent internal noise. This is because we apply independent external noise samples to each individual wavelet (and so measure the equivalent internal noise for each wavelet). In line with previous studies^13,16^, our ideal observer models predict that the equivalent internal noise should not depend on the number of samples available.

In the comparison with the controls we find that more external orientation noise is required to degrade performance in the contour task compared to the single wavelet task. This indicates that in contour integration there is a loss in the quality of the orientation information. On the other hand, the equivalent positional noise in the contour task is *lower* than that found for position discrimination with a single wavelet. Less positional noise must be added to affect the contour task compared to the control. This trade-off between orientation and position may arise from an intermediate stage where elongated receptive fields link adjacent wavelets. In that case, this trade-off should become more dramatic as the stimulus eccentricity increases^31^.

Another potential explanation for the increased equivalent orientation noise we find here can be found in *pedestal* masking studies that have been conducted on orientation variance discrimination^32,33^. These studies find a “dipper” function for their task, where small differences in orientation variance between groups of wavelets are easier to discriminate when both groups have a small pedestal variance (on top of which the increased variance to be discriminated is added). This facilitation effect can be explained by there being a “threshold” in the representation of orientation variance, perhaps to squelch the visual system’s internal noise. This may be relevant if observers identify the “good continuation” contour in this study by finding that in which the wavelet orientations have the smallest residual variance compared to the underlying contour. If this is the case then a task-dependent “squelching” of these variances would elevate the equivalent internal orientation noise. This would be consistent with previous reports of orientation discrimination threshold elevation within grating patches that form a contour^34^.

The novel equivalent noise approach to studying contour integration we present here bears some similarity to a previous task that has been used to measure the effects of perceptual learning on position discrimination^35,36^. The crucial difference however is that the task in that study was not designed to investigate contour integration, and could be solved by taking account of only position information. It is possible though that the same underlying mechanisms are responsible for performance in both tasks. When comparing the equivalent position noise between the two studies it is unsurprising that the positional noise found in our task is much higher (3 arcmin compared to 0.35 arcmin in Li *et al*.^36^). The participants in Li *et al*.^36^ could fixate the rows of wavelets as they pleased, and the stimuli were formed of wavelets of a higher spatial frequency (10 c/deg).

The comparison with Li *et al*.^36^ brings up the relationship between our study and those that have measured Vernier acuity. As both contour integration and Vernier acuity require discriminations to be made about the collinearity it is possible that there would be some overlap in how they are processed. It is thought that the contour integration process is carried out by lateral connections between neurones with adjacent receptive fields and collinear orientation preferences^37,38^. Vernier acuity, on the other hand, can be explained by the responses of neurones with oblique orientation preferences detecting the horizontal offset between two features^39–41^. Both capacities may represent cases where nonlinear interactions reshape and refine the response properties of local feature detectors based on the contextual modulation from other detectors. A general overview of these nonlinear interactions is presented from an interesting “geometric” perspective by Golden *et al*.^42^.

Our ideal observer modelling indicates that the fitted efficiency values *should* be different between the contour and control tasks (Table 5). In Tables 1–4 we present efficiency relative to these ideal observer values, which factors out this effect. We find that the human observers are relatively more efficient in the control tasks than the contour tasks, in terms of making use of all of the information that is available from the wavelets in the stimulus.This could be explained by there being 7× as many wavelets in the contour task for the observer to make use of. This may be too much information for the observers to handle efficiently. An alternative explanation would be that there are inherent inefficiencies in the way that the contour processing is performed. This question could be addressed by measuring efficiency relative to the ideal observer under different stimulus conditions.

**Table 5:** Fitted efficiency (*β*) of the noisy ideal observer models for the contour and control tasks. Shown here are mean log_2_ *β* values ± the standard deviation across the simulated internal noise levels shown in Fig. 6.

Model	log_2_ *β*_ideal_
Contour task, orientation noise	7.84 ± 0.04
Contour task, position noise	7.63 ± 0.04
Control task, orientation noise	0.16 ± 0.03
Control task, position noise	0.13 ± 0.02

Our novel contour task is a simplified and idealised approach to investigating how the visual system detects lines and edges in the outside world. Although the presented contours are formed of separate discrete elements, it is interesting that the percept is often of a continuous “joined up” contour. This modal completion can be contrasted with amodal completion, where contours implicitly join up past discontinuities. These situations are common in natural scenes^43^, and present a greater challenge to our ability to determine whether or not features belong to the same contour. The rules underlying amodal contour binding have been characterised by previous studies^44,45^. Future studies could explore how performance on our contour task can be used to investigate other limits on contour binding. For example, it should be possible to combine the performance limitations measured in this study with computational models of contour integration^2,9^, in order to predict performance in tasks with arbitrary contours presented alongside background elements. Beyond this, measurements of the equivalent internal noise for contour integration may be useful in conditions where we expect there may be deficits in visual processing. Such increases in neural variability have been reported in both autism and traumatic brain injury^46^.

## Methods

Procedures were approved by the Research Ethics Board of McGill University Health Centre, and carried out in accordance with the relevant regulations and guidelines. All subjects gave written informed consent. The experiment was programmed in Matlab using Psychtoolbox^47^. An Nvidia Quadro K5200 graphics card delivered a 10-bit contrast depth. Stimuli were presented on a gamma-corrected Flatron 915FT monitor. The mean luminance was 62 cd/m^2^ and the resolution 96 pixels per degree at the viewing distance used (77 cm). In each stimulus frame, there were contours placed in the four quadrants (top-left, top-right, bottom-left, and bottom-right) surrounding the fixation marker.

Contours were formed of seven log-Gabor wavelets^48^. The wavelets had a spatial frequency of 6 c/deg, cosine phase, and spatial frequency and orientation bandwidths of 1.6 octaves and ±25°. These were placed along a path defined by a cosine function. The *u* coordinates were *n* evenly spaced values across the length *m*

3 $${u}_{i}=m\times \frac{i-1}{n-1}-\frac{m}{2}\mathrm{.}$$

**Figure 5:**
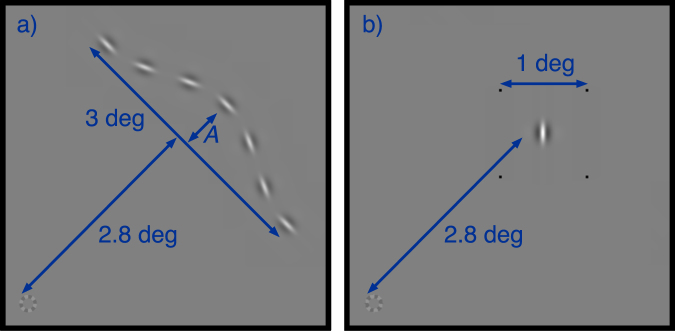
Stimulus design used in (**a**) the main study, and (**b**) the control study. Images show the stimuli presented to a single quadrant of the visual field. Similar stimuli were also shown (rotated about the fixation marker) in the other three quadrants.

In this study, the first and last wavelet of each contour were 3 degrees apart (*m* = 3 deg). The *v* coordinates, perpendicular to the *u* coordinates, depend on the amplitude of the curvature (*A*). The amplitude gives the deviation between the peak of the contour and the midpoint between the first and last elements (Fig. 5a). The coordinates are calculated as

4 $${v}_{i}={\rm{V}}({u}_{i},d,A,m)=d\times \frac{A}{2}\times (1+\,\cos \,[{u}_{i}\times \frac{2\pi }{m}]),$$

where the direction of curvature is controlled by *d* (which is either ±1 or −1). The orientations of the wavelets depend on whether we are generating a “valid” (*t* = 1) or “invalid” (*t* = 0) contour. For the valid case the orientations are consistent with the local path of the contour. For the invalid case the orientations are consistent with a contour curving in the opposite direction. We first calculate the local vector where ${u}_{i}^{^{\prime} }=2\delta$ and

5 $${v}_{i}^{^{\prime} }=\{\begin{array}{ll}{\rm{V}}({u}_{i}+\delta ,d,A,m)-{\rm{V}}({u}_{i}-\delta ,d,A,m), & {\rm{if}}\,t=1\\ {\rm{V}}({u}_{i}+\delta ,-d,A,m)-{\rm{V}}({u}_{i}-\delta ,-d,A,m), & {\rm{if}}\,t=0\end{array}$$

from which the wavelet orientation *θ*′ is found using the atan2 function

6 $${\theta }_{i}^{^{\prime} }={\rm{atan2}}({v}_{i}^{^{\prime} },{u}_{i}^{^{\prime} })\mathrm{.}$$

The coordinates (*u* and *v*) and angles *θ*′ are then rotated (by angle *p*) appropriate to the quadrant where the contour is being presented. In each quadrant the contours were presented at a tangent to a circle centred at fixation with radius 2.8 deg (see Fig. 5a). The $\bar{x}$ and $\bar{y}$ coordinates of each *i* ^th^ wavelet are given by

7 $${\bar{x}}_{i}=X(i,d,A,p)={u}_{i}\times \,\cos \,p-{v}_{i}\times \,\sin \,p\mathrm{.}$$

and

8 $${\bar{y}}_{i}=Y(i,d,A,p)={u}_{i}\times \,\sin \,p+{v}_{i}\times \,\cos \,p\mathrm{.}$$

and translated to the stimulus eccentricity. The orientations are simply

9 $${\bar{\theta }}_{i}=T(i,d,A,p,t)={\theta }_{i}^{^{\prime} }+p\mathrm{.}$$

This gives the final coordinates and orientations of the wavelets (the variables are barred because in the actual stimulus display there may be noise added to them). The contours were displayed at 60% contrast. The stimulus duration was 400 milliseconds.

We employed a 4-alternative forced-choice task. One random quadrant on each trial contained the valid contour and the other three contained invalid contours. After stimulus presentation, the observer pressed a key to indicate the quadrant with the valid contour. We used a method of constant stimuli design (128 trials of 6 stimulus levels, log-spaced between 2^−5.5^ and 2^−0.5^). Because the contour in each quadrant could be inflected inward or outward on each trial, we counterbalanced these conditions. Data were recorded to see whether either direction of curvature resulted in greater sensitivity.

For the orientation and position conditions we tested three noise levels, as well as testing without noise. For orientation, we applied a random rotation to every wavelet in the display

10 $${\theta }_{i}={\bar{\theta }}_{i}+{N}_{{\rm{ext}}},$$

where *N*_ext_ was drawn from a zero-mean normal distribution with standard deviation *σ*_ext_, determining the external noise level. The position noise was similar, with separate samples drawn to give random *x* and *y* coordinate offsets for each wavelet

11 $${x}_{i}={\bar{x}}_{i}+{N}_{{\rm{ext}}},$$

and

12 $${y}_{i}={\bar{y}}_{i}+{N}_{{\rm{ext}}}\mathrm{.}$$

For the contrast condition the noise samples determined the contrast of each wavelet. Pilot experiments showed no effect of contrast noise, so we tested only at a requested standard deviation of 32%. Due to clipping at 0% and 100% this resulted in an effective standard deviation of only 28%.

In the control experiments, observers performed orientation and position discrimination tasks. We replaced the contours with single 60% contrast wavelets (same contrast as our contours), flanked by black dots (Fig. 5b). These dots were 1.9 by 1.9 arcmins at 100% contrast, and remained clearly visible throughout the control task. The contour and control stimuli are presented centred at the same eccentricity (2.8 degrees of visual angle). Although the contour stimuli extend diagonally such that their ends will be at a slightly greater eccentricity, the most useful part of the stimulus for making the judgement in the task will be at 2.8 degrees. In these ways our control experiment is designed to allow comparisons to be made between the equivalent internal noise for the processing of wavelets in local versus contour tasks while minimising (as much as possible) the differences between the stimuli.

In the orientation control, observers indicated in which of the four quadrants was the wavelet rotated clockwise. This was performed with different levels of orientation noise applied to all four wavelets. For the position control the observer indicated which of the four wavelets had its position shifted to the right. This was done in different levels of 2D positional noise. Because performance on the control tasks was more variable, we tailored the stimulus levels (target rotation or shift) and noise levels for each observer. Fewer trials were collected in these control conditions. The plotted masking functions are based on data from 1,000–2,000 trials, compared to the >3,000 trials/condition for all observers in the contour task.

The data obtained from our experiments were fit by cumulative normal psychometric functions in Palamedes^49^. For the LAM analysis, the inverse of the function was used to calculate thresholds when *d*′ = 1. For the 4AFC task in this study this was at the 55.2% correct point of the psychometric function. Parametric bootstrapping was performed to generate a thousand bootstrap samples for each threshold. Bootstrapped estimates of the LAM parameters were obtained by fitting to sets of bootstrapped thresholds.

## Ideal Observer Modelling

We ran simulations of an ideal observer model for the contour task^20^. The ideal observer operates on the wavelet coordinates and orientations (and therefore does not predict any effect of wavelet contrast). It knows that there are a set of possible stimulus conditions that vary in curvature direction, amplitude and target location. The ideal observer also knows that the coordinates and orientations it receives will be noisy, and the standard deviation of that noise for each block. Briefly, the ideal observer uses the orientations and positions of the wavelets in the display to calculate the likelihood of each possible stimulus type given that information. It then responds on the basis of which target location is consistent with the most likely stimulus condition^50^.

On each trial the stimulus is defined by matrices of coordinates $\underline{\underline{{\rm{X}}}}$ and $\underline{\underline{{\rm{Y}}}}$, and orientations $\underline{\underline{{\rm{\Theta }}}}$. Each entry in the matrix (e.g. ${x}_{i,j}$) corresponds to the ${i}^{{\rm{th}}}$ wavelet (of the 7 per contour) in the ${j}^{{\rm{th}}}$ contour (of the 4 in our stimuli). From this the ideal observer calculates the likelihood of each stimulus condition. The conditions are defined by amplitude $A$, target location $l$, the curvature directions for each contour ${d}_{j}$ in $\underline{D}$ and global rotations (for the locations where the stimuli were presented) for each ${p}_{j}$ in $\underline{P}$. Log-likelihoods are summed across position and orientation

13 $$\mathrm{log}\, {\mathcal L} (A,l,\underline{D},\underline{P}|\underline{\underline{{\rm{X}}}},\underline{\underline{{\rm{Y}}}},\underline{\underline{{\rm{\Theta }}}},{\sigma }_{xy},{\sigma }_{\theta })=\,\mathrm{log}\,{ {\mathcal L} }_{xy}+\,\mathrm{log}\,{ {\mathcal L} }_{\theta }\mathrm{.}$$

The position likelihoods are calculated as the probability of obtaining the observed $x$ and $y$ coordinates under the considered stimulus condition with noise defined by the probability density function of the general normal distribution ${\rm{\varphi }}(x,\mu ,\sigma )$

14 $$\begin{array}{ccc}{\rm{l}}{\rm{o}}{\rm{g}}\,{{\mathscr{L}}}_{xy}(A,\mathop{D}\limits_{\_},\mathop{P}\limits_{\_}|\mathop{\mathop{{\rm{X}}}\limits_{\_}}\limits_{\_},\mathop{\mathop{{\rm{Y}}}\limits_{\_}}\limits_{\_},{\sigma }_{xy}) & = & {\sum }_{j}{\sum }_{i}\,{\rm{l}}{\rm{o}}{\rm{g}}\,\varphi [{x}_{i},X(i,{d}_{j},A,{p}_{j}),{\sigma }_{xy}]\\  &  & +{\rm{l}}{\rm{o}}{\rm{g}}\,\varphi [{y}_{i},Y(i,{d}_{j},A,{p}_{j}),{\sigma }_{xy}]\end{array}$$

where ${\sigma }_{xy}$ is the effective position noise combined across internal and external sources. The orientation likelihoods are calculated in a similar manner, however because orientation is circular we use the Von Mises distribution $\omega (x,\mu ,\sigma )$ instead of the normal, such that

15 $$\omega (x,\mu ,\sigma )=\frac{{e}^{\mathrm{(1/}{\sigma }^{2})\times \cos (x-\mu )}}{2\times \pi \times {I}_{0}(\mathrm{1/}{\sigma }^{2})}$$

where *I*_0_() is Matlab’s besseli function, used to give the modified Bessel function (of order 0), which scales the Von Mises probability density function so that it integrates to 1. In the implementation of the model the Von Mises function occasionally fails when the value of ${\sigma }_{\theta }$ is very small as there are terms in both the numerator and the denominator that become too large. In these cases we fall back on using the normal probability density function. In testing different versions of the ideal observer model we found that this only occurs when *σ*_*θ*_ < 2.2°. For values of *σ*_*θ*_ this small the difference between the two distributions results in disagreement on less than 0.01% of trials. With the Von Mises distribution, the orientation likelihood is calculated as

16 $${\rm{l}}{\rm{o}}{\rm{g}}\,{{\mathscr{L}}}_{\theta }(A,l,\mathop{D}\limits_{\_},\mathop{P}\limits_{\_}|\mathop{\mathop{{\rm{\Theta }}}\limits_{\_}}\limits_{\_},{\sigma }_{\theta })=\sum _{j}\sum _{i}\,{\rm{l}}{\rm{o}}{\rm{g}}\,\omega [{\theta }_{i},T(i,{d}_{j},A,{p}_{j},l==j),{\sigma }_{\theta }],$$

where *σ*_*θ*_ is the effective orientation noise combined across internal and external sources. Note also the $l==j$ comparison. This sets the value of $t$ in Eq. (5) to 1 or 0 depending on whether the ${j}^{{\rm{th}}}$ contour being evaluated is at the target location $l$. The log-likelihood is calculated for every combination of the possible amplitudes, target locations and curvature directions. The model then selects its response by finding the target location ($l$) for the most likely stimulus condition

17 $${\rm{response}}=\mathop{{\rm{argmax}}}\limits_{l\in \mathrm{\{1,2,3,4\}}}[\mathrm{log}\, {\mathcal L} (A,l,\underline{D},\underline{P}|\underline{\underline{{\rm{X}}}},\underline{\underline{{\rm{Y}}}},\underline{\underline{{\rm{\Theta }}}},{\sigma }_{xy},{\sigma }_{\theta })]\mathrm{.}$$

**Figure 6:**
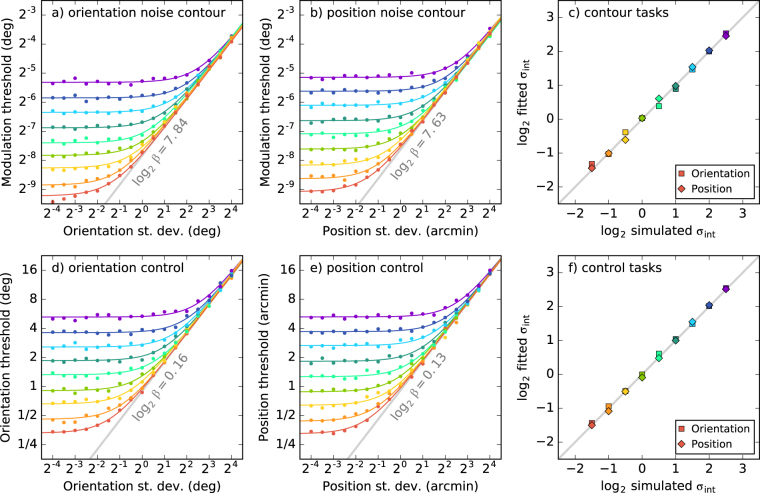
Predictions from noisy ideal observer models. The first two columns show simulated noise masking functions for a range of internal noise values (each with a different colour). The rightmost column shows how the fitted equivalent internal noise maps on to those simulated internal noise values (which can be compared to the diagonal unity line).

Although the ideal observer model usually does not feature internal noise, we ran simulations here with internal noise added to the model in order to demonstrate its behaviour. The predictions from this “noisy ideal observer” contour integration model are shown in Fig. 6a,b. Noise masking functions are shown for 9 simulated internal noise levels. The points show thresholds obtained by fitting psychometric functions (as above) to 6,000 simulated trials per point. As expected, the linear amplifier model (Eq. (2)) provides an excellent fit to these points. Figure 6c plots the fitted equivalent internal noise values against the simulated internal noise levels used to generate the data, showing that the LAM fitting recovers those values. The efficiency (*β*) parameters of the fitted LAM functions (Table 5) are very similar across the different internal noise levels.

For the control experiments, the ideal observer model was modified to consider only a single wavelet that is shifted either in its orientation or its position. For the orientation case the positions are irrelevant to the task, so only ${ {\mathcal L} }_{\theta }$ is considered. For the position case only ${ {\mathcal L} }_{xy}$ is considered. The noisy ideal observer predictions for the control experiments are shown in Fig. 6d–f, and mean efficiencies presented in Table 5.

### Data availability

The experiments were performed in accordance with the Declaration of Helsinki, and approved by the Research Ethics Board of McGill University Health Centre. All subjects gave written informed consent. Raw data are available from figshare, with doi: 10.6084/m9.figshare.4879580.
